# AMPK Function in Mammalian Spermatozoa

**DOI:** 10.3390/ijms19113293

**Published:** 2018-10-23

**Authors:** David Martin-Hidalgo, Ana Hurtado de Llera, Violeta Calle-Guisado, Lauro Gonzalez-Fernandez, Luis Garcia-Marin, M. Julia Bragado

**Affiliations:** 1 Research Group of Intracellular Signaling and Technology of Reproduction (SINTREP), Institute of Biotechnology in Agriculture and Livestock (INBIO G+C), University of Extremadura, 10003 Cáceres, Spain; davidmh@unex.es (D.M.-H.); anahl@unex.es (A.H.d.L.); violetacg@unex.es (V.C.-G.); lgonfer@unex.es (L.G.-F.); ljgarcia@unex.es (L.G.-M.); 2 Unit for Multidisciplinary Research in Biomedicine (UMIB), Laboratory of Cell Biology, Department of Microscopy, Institute of Biomedical Sciences Abel Salazar (ICBAS), University of Porto, 40050-313 Porto, Portugal; 3 Hormones and Metabolism Research Group, Faculty of Health Sciences, University of Beira Interior, 6200-506 Covilhã, Portugal

**Keywords:** AMP-activated protein kinase (AMPK), spermatozoa, motility, mitochondria, membranes, signaling, stress, assisted reproduction techniques

## Abstract

AMP-activated protein kinase AMPK regulates cellular energy by controlling metabolism through the inhibition of anabolic pathways and the simultaneous stimulation of catabolic pathways. Given its central regulator role in cell metabolism, AMPK activity and its regulation have been the focus of relevant investigations, although only a few studies have focused on the AMPK function in the control of spermatozoa’s ability to fertilize. This review summarizes the known cellular roles of AMPK that have been identified in mammalian spermatozoa. The involvement of AMPK activity is described in terms of the main physiological functions of mature spermatozoa, particularly in the regulation of suitable sperm motility adapted to the fluctuating extracellular medium, maintenance of the integrity of sperm membranes, and the mitochondrial membrane potential. In addition, the intracellular signaling pathways leading to AMPK activation in mammalian spermatozoa are reviewed. We also discuss the role of AMPK in assisted reproduction techniques, particularly during semen cryopreservation and preservation (at 17 °C). Finally, we reinforce the idea of AMPK as a key signaling kinase in spermatozoa that acts as an essential linker/bridge between metabolism energy and sperm’s ability to fertilize.

## 1. Introduction

**Figure 1:**
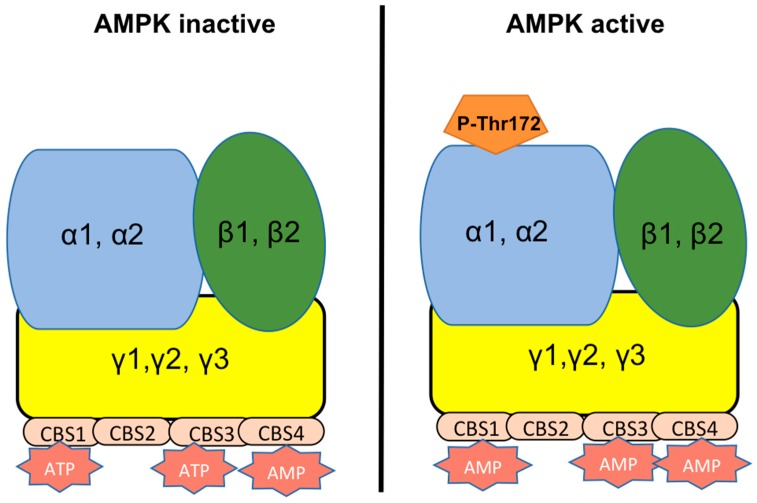
Structure of AMP-activated kinase (AMPK). AMPK is a heterotrimeric protein with 3 differents subunits: catalytic α (blue), scaffolding β (green) and regulatory γ (yelow), each one can be expressed as different isoforms, enabling distinct heterotrimeric combinations of AMPK. Regulatory subunit γ controls the activity of the α subunit through 4 tandem repeats of the motif CBS (cystathionine β-synthase) that bind adenosine nucleotides (AMP, ADP, ATP) with high affinity. AMPK remains inactive in presence of high levels of ATP, whereas during starvation or when ATP level decreases, CBS domains bind AMP, which favors the phosphorylation at Thr172 (catalytic α subunit) and subsequently leading to AMPK activation.

The AMP-activated protein kinase (AMPK) acts as a sensor molecule of cellular energy charge that maintains energy homeostasis at both the whole-body and cellular levels [1,2] by controlling key anabolic and catabolic pathways under different energy or stressful conditions. Thus, AMPK can be activated by physiological conditions, mediated by metabolic hormones, by different cell stress conditions, and also by pharmacological molecules [1,2]. This kinase is highly conserved in eukaryotic species and is ubiquitously expressed. Structurally, AMPK is a heterotrimeric protein including an alpha (α) catalytic subunit, a scaffolding beta (β), and regulatory gamma (γ) subunits, which are encoded by seven genes, allowing the formation of different heterotrimer combinations of α, β, and γ isoforms of AMPK (Figure 1).

Given its central regulator role in metabolism and its pleiotropic action in many cellular processes, AMPK has received enormous interest as a therapeutic target, and therefore, there has been a great effort to develop pharmacological activators. In addition, AMPK has been the focus of relevant works, although the majority of them have been conducted in somatic cells. However, the ability to preserve energy homeostasis under fluctuating extracellular nutrients or conditions that physiologically occur through the female reproductive tract is a necessary characteristic of spermatozoa. As mentioned before, the direct molecular connector between the substrate’s supply and energy requirement is AMPK. In 2012, two different research groups identified for the first time the presence of AMPK in mammalian spermatozoa [3,4]. Since then, interesting scientific efforts have been performed to elucidate the functions of AMPK in these male gametes in several species, including human, using different experimental approaches, including the use of α1AMPK knockout mice [3], and the pharmacological inhibitor and activators. To date, availability of AMPK inhibitors is very restricted and the most widely-used inhibitor of AMPK is the pyrazolopyrimidine derivate compound C or dorsomorphin (CC) that binds to the active site of AMPK and acts as an ATP-competitive inhibitor [5]. Very recently, a new pyrimidine derivative, SBI-0206965, has been discovered as a direct AMPK inhibitor with greater potency (40 fold) and lower kinase promiscuity than CC [6]. Regarding AMPK activators, great effort has been performed for the last few years in the development of direct small molecules and also in their mechanisms of action. The classical activator is the 5-aminoimidazol-4-carboxamide-1-B-D-ribofuranoside (AICAR), although its therapeutic use is not warranted in humans because of its elevated threshold for AMPK activation [7]. The first direct AMPK activator available to scientists was A769662, which activates AMPK both allosterically and by inhibiting AMPKα Thr172 dephosphorylation through binding at the ADaM site, and displays high specificity for complexes containing AMPK-β1 isoforms compared to AMPK-β2 [7]. An exhaustive review about other AMPK activators has been recently published [7] and includes cyclic benzimidazole derivatives (compound 991, PF-739, and MK-8722), the compound C2, the iminothiazolidione PT-1 and its optimized C24, the bi-quinoline JJO-1 and dihydroxyquinoline MT47-100, and recently indole- and indazole-acid-based AMPK synthetic activators, as well as two novel small activators, PXL770 and O304, which successfully completed phase I of clinical trials [7]. To date, the most clinically-used activator of AMPK is metformin and although its action was originally thought to be mediated by AMPK [8], likely through inhibition of AMP desaminase [9], it is currently established that many of metformin effects are AMPK-independent [10,11,12,13,14,15,16]. Thus, besides effects on glucose metabolism, metformin also directly inhibits complex I (NADH:ubiquinone oxidoreductase) of the mitochondrial electron transport chain [17,18] with obvious consequences in cell energy production. Overall, we believe that the modulation of AMPK activity would be an interesting tool for the future knowledge about exact roles of AMPK in mammalian spermatozoa, as well as for development of novel protocols for male fertility and/or sperm preservation in vitro.

In this review, we provide the current knowledge about AMPK function in spermatozoa under physiological and different extracellular conditions. AMPK activity is involved in the main physiological functions of mature spermatozoa, in particular in the maintenance of proper sperm motility, the integrity of sperm membranes, and the mitochondrial membrane potential, all of them adapted to the fluctuating medium of the female reproductive tract. Moreover, the intracellular signaling pathways leading to AMPK activation in mammalian spermatozoa are reviewed. We also discuss the role of AMPK in assisted reproduction techniques, particularly during semen cryopreservation and preservation at low temperature. Finally, we reinforce the idea of AMPK as a key signaling kinase in spermatozoa that acts as an essential connector between metabolism energy and sperm ability to fertilize.

## 2. Role of AMPK in Male Gonads and Spermatogenesis

Fertility can be affected by nutrition and energy metabolism; in this sense, AMPK plays an important role in the reproductive function, connecting, among other physiological regulators, the hypothalamus–pituitary–gonadal axis with energy balance [19]. Germ cells production in the testis occurs through spermatogenesis and terminates when the mature spermatozoa are released into the lumen of the seminiferous tubules. A proper energy balance is necessary for spermatozoa production in the testis, as well as for sperm function and quality after ejaculation. Nonetheless, only a few studies have focused on the role of AMPK in the control of male fertility.

In 2008, Towler et al. [20] indicated that kinases related to AMPK exert physiological functions in mammalian spermatozoa. Thereby, a shorter isoform of the tumor suppressor LKB1 (LKB1s) was shown to be mainly expressed in haploid spermatids [20] where it plays an essential role in spermiogenesis and in its fertilizing ability. *LKB1s* knockout mice have a high reduction in the number of mature spermatozoa in the epididymis, and the few spermatozoa produced are not motile and have abnormal head morphology, which causes sterile mice [20]. These data suggested that this variant of the LKB1 has a crucial role in spermiogenesis and fertility in mice. A new recent study has confirmed these results, where Kong et al. have shown that although LKB1s is the dominant isoform expressed in mice testis and plays a crucial role in spermiogenesis, the long isoform of the tumor suppressor LKB1_L_ was also required [21]. This group suggests an indispensable role of LKB1_L_ in spermatogonial stem cell maintenance and the cooperative regulations of both LKB1 isoforms in spermatids differentiation [21]. On the other hand, deletions of *TSSK1* and *TSSK2*, two members of testis specific TSSK family or Ser/Thr kinases, which belong to AMPK branch in the human kinome tree, cause infertility in chimera mice due to haploinsufficiency [22]. The above-mentioned studies point out that some AMPK-related kinases might play a key role in the spermatozoa production and function.

Male gonadal somatic cells include Sertoli (SCs) and Leydig cells (LCs). Proliferation and survival of somatic cells of the testis are crucial for fertility. In fact, testis size and sperm production are directly correlated to the total number of adult Sertoli cells. These cells play a central role in spermatogenesis by providing support and nutrition for spermatogenic cells; however, each SC nourishes a limited number of differentiating germ cells [23]. SCs maintain the blood–testis barrier, an essential feature of seminiferous tubules, which creates the proper environment for the spermatogenic process to be held [24]. It was demonstrated that maintenance of SCs cytoskeletal dynamics, polarity, and junctional communications are essential for fertility [25], and that disorders on their apical extensions, which direct migration of differentiating germ cells towards the lumen, leads to premature germ cell loss [26]. In 2012, Tanwar et al. showed the importance of the upstream AMPK kinase LKB1 signaling in rats SCs biology and spermatogenesis. They demonstrated that aberrant AMPK-mTOR signaling causes disruption of SCs polarity and spermatogenesis [27]. In the same year, Riera et al. demonstrated that the follicle-stimulating hormone (FSH) regulates SCs proliferation with the participation of the PI3K/Akt/mTORC1 pathway and that AMPK activation may be involved in the detention of proliferation by, at least in part, a decrease in mTORC1 signaling and an increase in cyclin-dependent kinase inhibitor (CDKI) expression [28]. Thus, well-coordinated AMPK-mTOR signaling is essential for SCs functions and survival in rats [27,28]. According to a recent study, heat treatment could reversibly perturb the expression of tight junction proteins in immature porcine SCs by inhibiting the AMPK signaling pathway [29]. The use of the AMPK activator AICAR can affect adhesion molecule expression and influences junction complex integrity in rat SCs, as it has been described in 2010 by Galardo et al. [30]. This group also suggested an important role of AMPK in modulating the nutritional function of SCs. They demonstrated that AMPK stimulation with AICAR increased lactate production and glucose transport [31]. On the other hand, the inactivation of α1AMPK in mice SCs reduces the expression of mitochondrial markers (cytochrome c and PGC1-α) and the ATP content, and conversely, increases the lactate production and lipid droplets [32]. Specific deletion of the α1AMPK gene in mice SCs resulted in a 25% reduction in male fertility associated with abnormal spermatozoa with a thin head. Testes showed no clear alterations of morphology or modification in the number of SCs in vivo, but a deregulation in energy metabolism in SCs was observed [32]. These authors suggested that deregulation of the energy-sensing machinery through disruption of α1AMPK in SCs leads to a reduction in the quality of germ cells and fertility [32]. These results supported those obtained by Tartarin et al. performed in mice, where the indirect AMPK activator metformin decreased testosterone production in vitro and reduced the testicular size and the population of SCs in vivo [3]. The involvement of AMPK activity has also been studied in SCs under different stimuli/conditions and from other species such as boar. Thus, Jiao et al., reported that 17β-estradiol inhibited immature boar SCs viability and cell cycle progression by activating the AMPK signaling pathway. Furthermore, they showed that AMPK activation by AICAR inhibited SCs viability, while the AMPK inhibitor compound C attenuated the effects of 17β-estradiol on SCs [33]. Besides these findings in mammalian testes, it has been reported that AMPK activity also exerts regulatory functions in avian gonads, as metformin treatment also reduced SCs proliferation and increased lactate secretion in chickens [34].

The precise composition of the extracellular environment of germinal cells in the seminiferous tubules remains unknown, but it is well established that SCs secrete molecules that are crucial for the spermatogenic process [35]. In this sense, SCs secrete proteins and cytokines, which participate in the control of spermatozoa movement from the testis to the epididymis, and in the control of the pH of the seminiferous fluid [36]. During spermatogenesis, lactate is the preferred substrate of spermatocytes and spermatids. Lactate production from glucose in SCs is an important point for the control of spermatogenesis [37]. Recently, it has been demonstrated that AMPK reduces heat-induced lactate secretion by decreasing the expression levels of the glucose transporter GLUT3, LDHA, and MCT1 [38]. This work also suggested that AMPK is a negative regulator of heat treatment-induced lactate secretion in cultured boar SCs [38].

**Figure 2:**
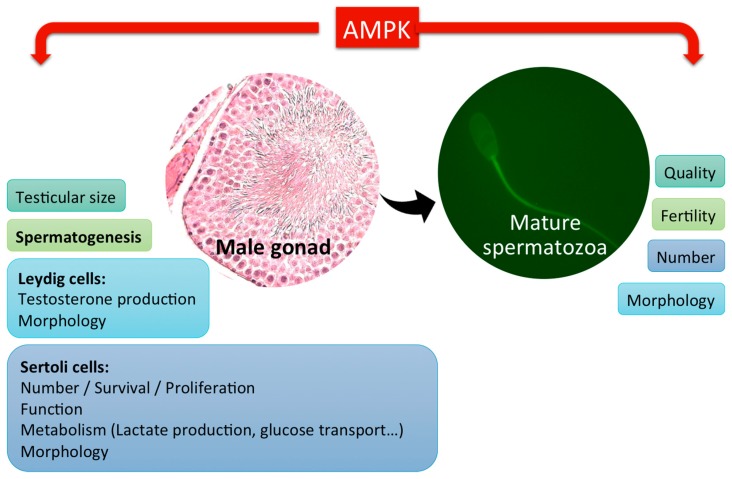
AMPK functions in male gonads and spermatozoa production. AMPK activity is involved in the control of the male gonad function, spermatozoa production and quality by its action also on the male gonadal somatic cells (Sertoli and Leydig cells).

It has been demonstrated that AMPK is involved in the regulation of spermatozoa quality through its action, not only on the proliferation of SCs, but also in another type of testicular somatic cells, Leydig cells (LCs), (Figure 2) which are responsible for producing testicular androgens, such as testosterone, for the paracrine regulation of spermatogenesis within the testis in adult life [39].

Steroid hormones regulate essential physiological processes, thus, testosterone is also responsible for the differentiation and development of male reproductive organs, as well as for maintaining secondary sex characteristics and sexual function. Within the testis, the functions of the LCs are mainly controlled by the hypothalamus–pituitary axis. An *α1AMPK* knockout mice model has been studied by Tartarin et al. [3]. The male α1AMPK^−/−^ mice have high levels of testosterone due to hyperactive LCs [3]. Indeed, the LCs of these animals have an increased volume, an altered endoplasmic reticulum area, a high intratesticular cholesterol concentration, and a greater expression of proteins involved in steroid production [3]. In agreement with this data, Abdou et al. identified AMPK as a molecular rheostat that actively represses steroid hormone biosynthesis to preserve cellular energy homeostasis and prevent excess steroid production in LCs [40]. Recently, the role of AMPK in steroidogenesis has also been investigated in vivo in ovine testis by Taibi et al. These authors report that active AMPK is expressed in this gonad, and more importantly, they demonstrated that AMPK activity is regulated by nutritional status [41]. However, to date, the expression patterns of the different AMPK isoforms in spermatozoa are unknown. One of the most recent studies revealed that resveratrol enhanced AMPK phosphorylation and may exert its cytoprotective role against oxidative injury by the activation of autophagy via AMPK/mTOR pathway in LCs [42]. Beside mammals, the effect of metformin has also been studied in avian species; thus in 6-week-old chickens, metformin induces a decrease in testosterone levels and reduces testis size [34].

To date, AMPK has also been studied in male gonads from other mammal species such as rats [28,43,44,45,46,47,48], mice [49,50,51,52,53,54,55], lambs [56], monkeys [48], and humans [57]. Taking into account all the aforementioned articles, it can be concluded that the AMPK pathway is involved in the control of the male gonad function, spermatozoa production, and quality by its action also on the male gonadal somatic cells and point out AMPK as a novel gatekeeper of steroidogenesis and a target for modulating steroid hormone production. Therefore, AMPK appears as a key signaling protein for the spermatozoa and male fertility control.

## 3. Localization of AMPK in Mammalian Spermatozoa

The presence of the AMPK protein in mature spermatozoa was demonstrated independently in 2012 in two mammalian species, boar [4] and mouse [3]. Later, AMPK protein localization has been confirmed in mouse cauda epididymal spermatozoa [58] and also in spermatozoa from stallions [59,60], rats [61], and very recently, goats [62] and humans [63,64]. Immunolocalization techniques reveal that AMPK localization in spermatozoa slightly varies between species. AMPK is localized at the entire acrosome and in the midpiece of flagellum in boar spermatozoa [65]. Interestingly, in this species, when AMPK becomes phosphorylated at Thr172 (active) is specifically restricted to the most apical part of the acrosome and to the subequatorial segment, remaining in the midpiece of the flagellum [65]. In human spermatozoa, AMPK is localized at the entire acrosome, the midpiece and along the tail of the flagellum [63,64], whereas its active form is found only at the most apical part of the acrosome and along the entire tail, with very slight amounts in the post-acrosomal region and in the midpiece of the flagellum [63]. In stallion spermatozoa, active AMPK is mainly detected in the subequatorial region in the head and in the principal piece of the tail [59]. AMPK localization has also been studied in spermatozoa from avian spermatozoa [66]. Thus, in chicken spermatozoa, AMPK is present in the acrosome, the intermediate part, and the whole flagellum, whereas active AMPK is mainly localized in the flagellum and the acrosome [66].

## 4. Functions of AMPK in Mammalian Spermatozoa

### 4.1. Role of AMPK in the Regulation of Spermatozoa Motility

**Figure 3:**
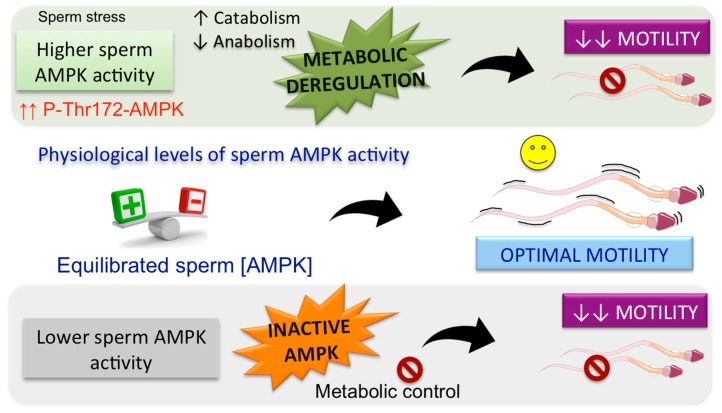
Different AMPK activity levels control spermatozoa motility. *In vivo*, during the spermatozoa transit trough the female reproductive tract, fluctuating levels of AMPK activity might occur in spermatozoa depending on the sperm energy demands. A physiological range of energy-charge regulated levels of AMPK activity is essential to accomplish optimal sperm motility that is adapted to the fluctuating extracellular conditions within the female reproductive tract. Up or down fluctuations of sperm AMPK activity cause a negative role in mammalian sperm motility.

Sperm metabolic plasticity, and particularly the capacity to switch substrates and also to respond to stimuli, renders a transcendent adaptation to sperm extracellular medium fluctuations, which might include exhausted energy, high-energy demand, and low oxygen availability. AMPK plays a central role in the regulation of energy metabolism homeostasis [1,2], and therefore, this kinase has recently emerged as a new signaling pathway that exerts a necessary control of sperm function. The specific and primordial function of spermatozoa fertilization is highly dependent on the proper sperm motility. Motility is a characteristic sperm functional process that is tightly regulated in response to the spermatozoa environment and to the subsequent changes in energy demands during its transit trough the female reproductive tract. AMPK involvement on sperm motility has been demonstrated by different experimental approaches that were independently performed in two mammalian species, boar [4] and mice [3]. Thus, transgenic mice lacking the catalytic subunit α1 gene (*α1AMPK* knockout) have a dramatic reduction in sperm motility and curvilinear velocity [3]. Similarly, a pharmacological approach in boar spermatozoa demonstrates that AMPK inhibition by CC significantly decreases the percentage of motile spermatozoa, the sperm curvilinear velocity and subsequently diminishes the percentage of rapid spermatozoa (with average velocity >80 μm/s), also affecting other motility parameters and coefficients evaluated specifically using a computer-assisted sperm analysis system [4]. Later, the important regulatory role of AMPK in spermatozoa motility was demonstrated for other mammalian species such as human [63,64], rat [61], stallion [60], and goat [62]. This clear role of AMPK in sperm motility suggests that this kinase might phosphorylate downstream substrates including proteins of the axoneme or in other related structures that are indispensable for sperm flagellar motility, as it has been previously demonstrated for the AMPK related kinase TSSK2 [67]. Thus, sperm TSKK2 phosphorylates the axoneme protein SPAG16L (sperm associated antigen 16) in vitro, a protein necessary for flagellar motility in mouse spermatozoa [68]. Surprisingly, by using a specific pharmacological AMPK activator, A769662 [69], it has been shown that an increase in AMPK activity above physiological levels also adversely influences sperm motility in two mammalian species, boar [70] and human [71]. Therefore, it has been proposed [70,72] that either up or down fluctuations of sperm AMPK activity (away from energy charge-regulated physiological levels) cause a negative role in mammalian sperm motility. A range of physiological levels of AMPK activity is essential to accomplish optimal sperm motility (Figure 3) that must adapt to the fluctuating extracellular conditions that spermatozoa can be physiologically exposed to [19,70,72].

In vivo, during the sperm transit trough the female reproductive tract, fluctuating levels of AMPK activity might occur in spermatozoa depending on the sperm energy demands. Whenever environment-depending demands of energy lead to a lower sperm AMPK activity that falls below the so-called physiological range, inactive AMPK is unable to adjust sperm metabolic pathways to control and maintain energy levels required for spermatozoa motility, which is negatively affected, as demonstrated in several species [3,4,62,63]. The proposal that a physiological range of AMPK activity is necessary for appropriate sperm motility is firmly supported by a study performed in transgenic mice lacking the catalytic subunit α1 gene [α1AMPK knockout (KO)] which presents a great reduction in sperm motility and curvilinear velocity [3]. Whenever environment-depending demands of energy lead to a sustained sperm AMPK activity raised above the physiological range (i.e., sperm stresses), the sustained AMPK activity would lead to a deregulation of sperm metabolism caused by a prolonged stimulation of ATP-generating catabolic pathways and by a sustained inhibition of ATP-consuming anabolic pathways (Figure 3). Thus, the deregulation of sperm metabolic pathways is not suitable to accomplish proper sperm motility under any extracellular conditions, as demonstrated in A769662-treated spermatozoa from boars [70] and humans [71]. In accordance with these findings, it has been demonstrated that spermatozoa exposure to stress stimuli, such as pollutants like hydrogen sulfide and/or ammonia, which lead to AMPK activation, causes a decline in boar spermatozoa motility [73]. In addition, this proposal is also supported by recent results obtained using a more physiological method of arresting the motility activation of rat caudal spermatozoa by isolating and lowering temperature (0–2 °C) without freezing [61]. AMPK activity in rat quiescent spermatozoa is higher than in motile spermatozoa [61], which supports the idea that AMPK is effectively regulating sperm motility by allowing these male gametes to adapt to extracellular-driven changes in sperm energy charge. Thus, the potent activation of AMPK in rat quiescent spermatozoa guarantees the most efficient pathways of energy metabolism and promotes their survival under a low energy-starved status with very limited energy resources [61]. Under these circumstances of an elevated AMPK activation, rat quiescent spermatozoa are not motile. When sperm substrates/stimuli become available during the initiation of sperm motility after ejaculation, AMPK activity levels diminish compared with quiescent spermatozoa [61], although they are still detectable and likely remain at the so-called physiological range of AMPK activity in rat spermatozoa. This female tract environment-driven shift in AMPK activity to a physiological level is necessary to adjust the proper sperm motility under these new requirements in energy demand [61].

However, very recently, one study found that other AMPK activators, such as AICAR and metformin, improve progressive motility in goat spermatozoa [62]. It is important to mention several arguments that might account for the different results found in this species: (a) The increase in phospho-AMPK levels obtained by AICAR and metformin in goat spermatozoa is very slight [62] compared with the high AMPK activity levels observed in boar [70] or rat [61] that lead to inhibition of sperm motility. Moreover, these two activators, AICAR and metformin, fail to activate AMPK in spermatozoa from other species such boar [70], where only A769662 was found to be effective activating AMPK. In addition, metformin also fails to effectively increase AMPK phosphorylation in human spermatozoa [71]. (b) It is reasonable to assume that besides great differences in the intensity of AMPK activation and the differences attributable to spermatozoa from distinct species, the specificity and effectiveness of each pharmacological activator should be kept in mind, also for further studies. This review tries to summarize the current knowledge about the role of AMPK activity in the regulation of sperm motility, and it seems clear that future research in this area is needed to elucidate the exact molecular mechanism by which AMPK exerts this regulation in sperm cells and also to discover AMPK protein substrates that belong to the intracellular signaling pathways controlling sperm motility.

### 4.2. Role of AMPK in the Regulation of Spermatozoa Mitochondrial Activity

As an energy regulator protein, AMPK has additionally been pointed out as important pathway that contributes to the maintenance of the mitochondrial membrane potential (∆Ψm) in spermatozoa [3,65]. Different experimental approaches designed to inactivate sperm AMPK cause a decrease in sperm ∆Ψm in different species: goat [62], boar [65], stallion [60], and α1AMPK knockout mouse [3]. Besides a fall (50%) in sperm ∆Ψm, α1AMPK knockout (KO) mice exhibited a lower number of mitochondria and had a significant lower consumption (60%) of basal oxygen [3]. Furthermore, a short up-activation of sperm AMPK using A769662 prevents the decrease in the number of Ca^2+^ and bicarbonate-stimulated spermatozoa presenting high ∆Ψm in boar [70], whereas it has no significant effect in ∆Ψm of human spermatozoa [71]. However, the indirect activator of AMPK resveratrol significantly increased ∆Ψm in frozen–thawed human spermatozoa [64]. In mouse fresh spermatozoa, another indirect activator of AMPK metformin causes a concentration-dependent decrease of ∆Ψm [74]. Thus, AMPK activity seems to be involved in the regulation of sperm ∆Ψm, although there are differences between species, the (direct or indirect) pharmacological compound used to activate AMPK and the cell status of spermatozoa (fresh or frozen–thawed). In any case, the effect of AMPK in sperm ∆Ψm, when demonstrated, is clearly dependent on sperm extracellular stimuli [70,72], pointing out AMPK as a metabolic checkpoint by integrating stimuli-induced signaling with sperm metabolism [70]. Therefore, it has been proposed that AMPK activity (at specific levels) is essential to maintain sperm mitochondrial membrane potential [70,72]. Thus, AMPK contributes to modulate the mitochondrial membrane potential according to extracellular stimuli or conditions-derived sperm energy, and subsequently promotes the proper sperm motility, at least in some species. The involvement of AMPK in the modulation of sperm ∆Ψm is further supported by the localization of important amounts of active AMPK (Thr172 phosphorylated) at the midpiece of mammalian spermatozoa where mitochondria are located, as it has been demonstrated in boar [65], goats [62], stallions [60], and humans [63].

### 4.3. Role of AMPK in the Regulation of Spermatozoa Membranes

Mammalian spermatozoa need a tight modulation of energy also to maintain cellular structure, stability, and physiology of membranes during fluctuating extracellular conditions within the female reproductive tract that ultimately allow them oocyte fertilization [75,76]. An important factor that contributes to the correct function of spermatozoa is the degree of lipid organization of their plasma membrane. In fact, the energy charge-sensor kinase AMPK plays a role in the maintenance of sperm plasma membrane fluidity and lipid organization as it has been demonstrated in some species such as boar [65] and goat [62]. Thus, experiments using either an AMPK inhibitor (CC) in boar [65] and goat spermatozoa [62], or the activator A769662, demonstrated a significant increase in plasma membrane lipid disorganization [70]. Importantly, this effect of AMPK is clearly dependent of the stimuli present in the extracellular medium, as sperm plasma membrane disorganization is ≈3-fold higher when boar spermatozoa are incubated in the presence of capacitating stimuli (Ca^2+^, bicarbonate, and serum albumin) than in a stimuli-free medium [65]. All together, these findings suggest that specific physiological levels of AMPK play a regulatory role in the preservation of the proper lipid organization and fluidity in sperm plasma membrane according to extracellular stimuli [65,77]. Furthermore, physiological levels of bicarbonate cause a quick collapse of the asymmetry of the sperm plasma membrane attributable to the scramblases activation, which translocate phospholipids such as phosphatidylethanolamine and phosphatidylserine (PS) outward of plasma membrane [78]. The PS externalization evidences plasma membrane scrambling which physiologically takes place in important sperm processes. Interestingly, AMPK inhibition in spermatozoa incubated in the presence of bicarbonate result in a significant inhibition of the outward exposure of PS in plasma membrane, suggesting that inhibition of AMPK, at least over a short time (4 h), could be causing a downstream inhibition of scramblases activity [65]. Moreover, an enhanced AMPK activity (for 24 h) induces a significant PS externalization in boar sperm plasma membrane [70]. These functional consequences of AMPK in boar sperm plasma membrane, which are lipid disorganization and the PS translocation, are presumably taking place at the plasma membrane surrounding the most apical acrosome region, where most of active phospho-Thr172 AMPK is restricted in this specie [65]. The involvement of AMPK in boar sperm membranes organization is supported by the finding that AMPK is downstream of the cAMP/PKA pathway [79], which is an essential regulatory pathway for the lipid architecture of the sperm plasma membrane [80,81].

Besides the regulatory role at the sperm plasma membrane, AMPK also regulates acrosome membrane integrity under capacitating conditions [65], which is additionally supported by the fact that high levels of active phospho-Thr172-AMPK are situated at this part of acrosome under physiological conditions [65]. By using different experimental approaches it has been demonstrated that any oscillation either up (A769662) or down (CC) of the AMPK activity outside of physiological levels lead to a loss of the outer acrosome membrane integrity [65,70]. In summary, physiological sperm AMPK activity is also essential to keep the integrity of acrosome membrane at a level suitable to the fluctuating extracellular conditions at which spermatozoa are physiologically exposed.

### 4.4. Role of AMPK in the Regulation of Spermatozoa Acrosome Reaction

Mammalian spermatozoa need to acquire the ability to reach the oocyte, penetrate the cumulus oophorus, and to bind to the zone pellucida of the oocyte, which subsequently triggers the acrosome reaction that leads to egg fertilization [76]. The acquisition of these spermatozoa functional competences occurs through important physiological and biochemical modifications that are collectively named sperm capacitation. Spermatozoa processes that necessarily must occur during capacitation are dependent on the energy levels of this gamete [82,83]. Therefore, it might be reasonable to assume that the energy charge-sensitive AMPK should be somehow involved in the acquisition of these sperm processes, although to date, few works have considered this issue. In fact, the activity of the energy charge-sensitive AMPK is regulated under capacitating conditions (including Ca^2+^, bicarbonate, and serum albumin) in boar spermatozoa [65]. The inhibition of AMPK activity significantly reduces the integrity of the acrosomal membrane in a boar sperm-capacitating medium, whereas it has no effect in a stimuli-free medium [65]. These results indicate that AMPK is involved in the regulation of sperm events that take place at acrosome membrane during capacitation, at least in boar spermatozoa. However, although AMPK contributes to the maintenance of acrosome membrane integrity, is not likely involved in the sperm process of acrosome reaction, as the inhibition of AMPK in capacitated boar spermatozoa did not affect the acrosome reaction triggered by the calcium ionophore A23187 [65]. This lack of effect of AMPK activity in the acrosome reaction has been demonstrated also in fresh mouse spermatozoa using the indirect activator of AMPK metformin [74]. Moreover, AMPK up-activation does not affect the integrity of the sperm acrosome membrane. Thus, it has been demonstrated that the activator A769662 has no measurable effect in the integrity of the acrosome membrane in human [71] or boar spermatozoa, independently of stimuli present in the medium [79]. However, the effect of A769662 in boar seems to be dependent on the stimulation time as a sustained AMPK activity over 24 h causes a significant loss of outer acrosome membrane integrity [79]. In contrast to mammals, few works performed in avian sperm show that AMPK activity plays a role in the acrosome reaction [66,84]. These differences between mammals and avian spermatozoa highlight the importance of further research about the functional role of AMPK in the acrosome reaction. To date, it can be concluded that in parallel to modulate physiological changes (lipids organization) that occurs at the mammalian plasma membrane during the sperm capacitation, AMPK activity contributes to the maintenance of the outer acrosome membrane integrity, where a majority of phospho-Thr172-AMPK active is localized at physiological conditions [4].

## 5. Signaling Pathways Leading to AMPK Activation in Spermatozoa

**Figure 4:**
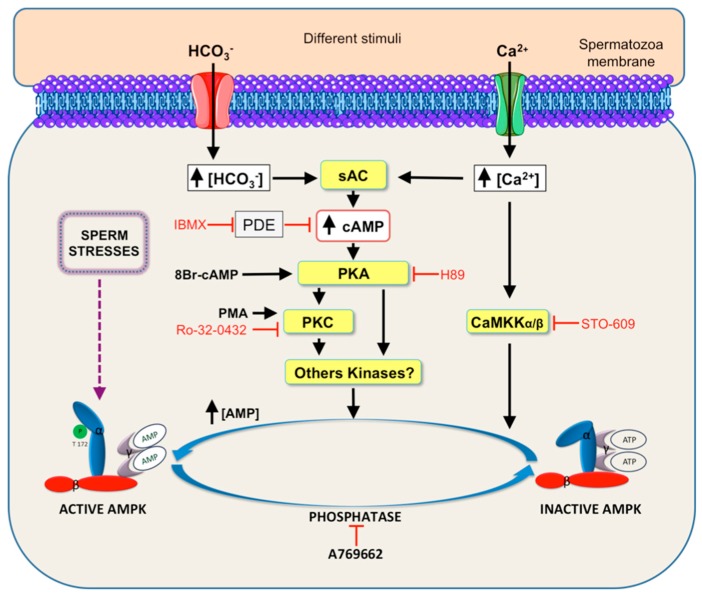
Signaling pathways underlying the regulation of AMPK activity in mammalian spermatozoa. This Figure summarizes the intracellular kinases and mechanisms that have been demonstrated to be involved in AMPK activation by Thr172 phosphorylation: soluble adenylyl cyclase (sAC), cAMP, protein kinase A (PKA), protein kinase C (PKC), intracellular Ca^2+^, calcium and calmodulin kinase kinases α/β (CaMKK α/β) and different types of cell stresses detailed in the text (absence of Ca^2+^ by BAPTA-AM, hyperosmotic stress, inhibition of mitochondrial activity). Pharmacological inhibitors of different AMPK upstream kinases (H89, IBMX, STO-609 and Ro-0432) are also indicated with red lines.

The function of AMPK as a key regulatory molecule of the essential processes that contribute to the spermatozoa function of fertilization has been reviewed above. Therefore, an important issue in spermatozoa physiology is to elucidate the signaling pathways leading to AMPK activity (Figure 4). Several works using different experimental approaches demonstrate that soluble adenylate cyclase (sAC), cAMP [4], and cAMP-dependent protein kinase (PKA)-mediated pathway lie upstream of AMPK activity in boar spermatozoa [65,79].

The sperm cAMP/PKA pathway might promote AMPK activation through its upstream kinase LKB1, as it occurs in somatic cells [85], where it is reported that LKB1 can be directly phosphorylated at Ser431 by PKA in response to activation of adenylate cyclase by forskolin [86,87] or IBMX [86]. It has been demonstrated that the short splice variant of LKB1_S_ is highly expressed in haploid spermatids in mice testis [20], where it is critically involved in spermiogenesis and fertility [88]. The knocking out LKB1_S_ in male mice causes important reproductive consequences: sterility, a marked decrease in the number of mature spermatozoa, spermatozoa showing an abnormal head morphology, and are non-motile. An increase in cAMP concentration might also lead to AMPK activation trough cAMP degradation to AMP by phosphodiesterases, as occurs in somatic cells [89]. Thus, any sperm stimulus that increases intracellular cAMP levels could promote AMPK activation either (i) by direct (allosteric) activation of PKA, or (ii) by indirect activity of phosphodiesterases, which increase AMP levels that allosterically activate AMPK, or (iii) by both pathways.

The intracellular messenger Ca^2+^ is also an essential regulator of spermatozoa functional processes. Intracellular Ca^2+^ activates the specific sperm sAC and its downstream signaling through PKA, therefore potently activates AMPK in boar spermatozoa under physiological conditions [65,79]. However, spermatozoa from the same species under conditions considered high extracellular Ca^2+^ concentrations (>3 mM), sperm AMPK phosphorylation decreases [90]. The involvement of Ca^2+^ as an upstream regulator of AMPK activity has also been demonstrated in avian spermatozoa, as Ca^2+^ entry via store-operated Ca^2+^ channel (SOC) activates AMPK [84]. Moreover, Ca^2+^ can also lead to the activation of boar sperm AMPK through the activation of Ca^2+^-calmodulin dependent kinase kinases II and CaMKKα/β as demonstrated in spermatozoa from boar [79] or chicken [91], which lie upstream of AMPK in somatic cells [92,93].

Sperm AMPK phosphorylation is also stimulated by direct activation of PKC with phorbol 12-myristate 13-acetate (PMA), whereas the PKC inhibitor Ro-32-0432 inhibits HCO_3_^−^ and Ca^2+^-induced AMPK activation in boar spermatozoa [79]. These findings indicate that at least one isoform of PKC is upstream of sperm AMPK. Several isoforms of PKC have been identified in mammalian spermatozoa: PKCα and PKCβI in bovines [94], PKC-zeta in hamsters [95], and mouse spermatozoa [96]. It is therefore plausible that some of these PKC isoforms might exert in spermatozoa a similar function than in somatic cells by phosphorylating LKB1 and subsequently leading to AMPK activation. Thus, it has been demonstrated that PKC-zeta phosphorylates LKB1 at Ser 307 [97] and PKCζ phosphorylates LKB1 at Ser 399 [98]. An alternative explanation describing the pathway by which PKC is upstream of sperm AMPK activity is based on the fact that PKC activity lies downstream of PKA in the control pathway of boar sperm motility [99]. Previously, Harayama and Miyake (2006) demonstrated that cAMP/PKA signaling can induce the activation of calcium-sensitive PKCs, which are responsible for boar sperm hyperactivation [100]. Thus, it is proposed that another PKC isoform(s) besides PKC-zeta, which is not calcium sensitive, could likely be involved in AMPK activation, at least in response to an elevation of cAMP levels in boar spermatozoa [79].

In addition to the mentioned physiological mimicking conditions, AMPK becomes markedly activated in boar spermatozoa under different stimuli considered to be cell stress (Figure 1), such as inhibition of spermatozoa mitochondrial activity by blocking electron transport chain and sorbitol-induced hyperosmotic stress [79]. In somatic cells, cell stress-induced AMPK activation can be mediated by (i) an increase in AMP levels, and/or (ii) reactive oxygen species ROS generation that act as signaling molecules to activate AMPK [101] through LKB1 and CaMKKs pathways. Surprisingly, the incubation with the intracellular calcium chelator BAPTA-AM in a Ca^2+^-free medium leads to a strong increase in AMPK activity [79], which may be mediated through an increase in nitric oxide NO· production. In this sense, de Lamirande et al. (2009) demonstrated in human sperm that BAPTA-AM promotes the production of a reactive oxygen specie, the nitric oxide NO· [102]. Accordingly, AMPK activation is also directly influenced by cellular redox status in somatic cells, as H_2_O_2_ activates AMPK through oxidative modification of cysteine residues in the AMPKα subunit [103]. An alternative or simultaneous explanation is that NO· produced by BAPTA-AM in boar spermatozoa might interacts with the cAMP pathway, as it occurs in human [102], leading to AMPK activity.

Moreover, other different types of stimuli that activate AMPK in mammalian spermatozoa have recently been described. Thus, pharmacological compounds, such as the anti-diabetic drug rosiglitazone, increase AMPK phosphorylation in stallions [60]. Also, some toxicant compounds that cause a marked reduction in sperm motility, such as the heavy metal cadmium, a major environmental toxicant, affected AMPK activity in mouse spermatozoa [104], or the air pollutants hydrogen sulfide and ammonia that activated AMPK activity in boar spermatozoa [73]. Additionally, the common natural mycotoxin ochratoxin A triggers AMPK activation to cause a clear decrease in boar sperm motility [105]. Based on all above-mentioned works, the exact molecular mechanisms involved in the signaling pathway(s) triggered by a variety of stimuli that lead to AMPK activity as well as the identity of AMPK downstream targets that ultimately control sperm function, undoubtedly deserve future investigations.

## 6. Role of AMPK during Assisted Reproduction Techniques: Semen Preservation

Assisted reproduction techniques (ART) include a wide range of technologies used to improve the chances to achieve pregnancy after the collection and handling of oocytes, sperm, and embryos in in vitro conditions. Nowadays, around 1.5 million human ART cycles are performed each year worldwide, with a reported 333,000 babies born. Data from human spermiograms show a decrease of sperm quality in the last decades [106,107,108], and as a consequence, an increase of the use of ART, such as intracytoplasmatic sperm injection (ICSI), sperm cryopreservation, in vitro fertilization (IVF), or the use of spermatozoa from testicle biopsies are the most commonly used ART. In the animal field, the use of ART is mostly aimed to quickly spread genetic material of selected animal to preserve gametes from endangered species, also to reduce disease transmission risk or to increase animal breading. This current and future reality leads to an increasing ART use and develop of new technical strategies to overcome fertility problems.

### 6.1. Why AMPK Protein Is Important in ART?

**Figure 5:**
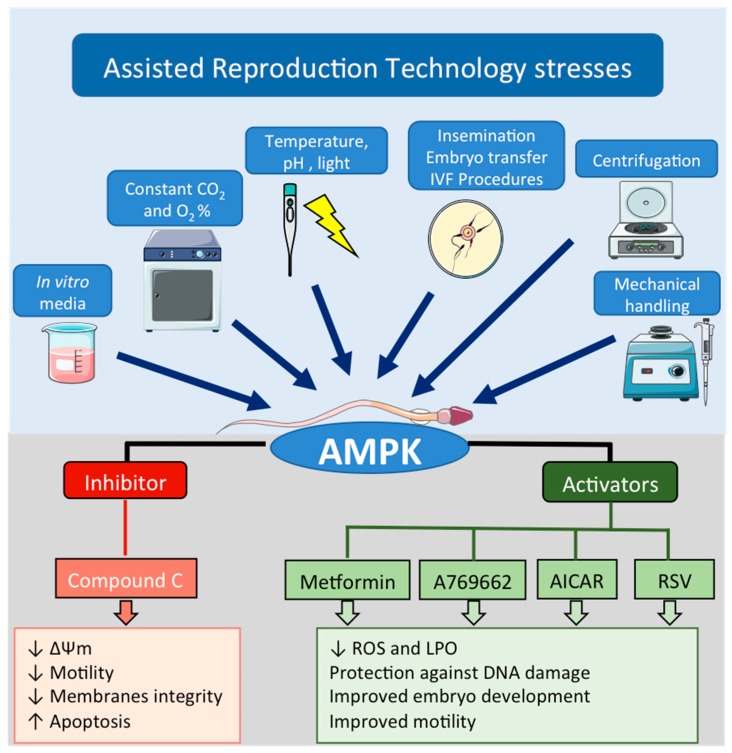
Effects of the modulation of AMPK activity (up or down) in spermatozoa during Assisted Reproduction Techniques (ART), including In Vitro Fertilization (IVF). During ART, spermatozoa are subjected to different stress conditions (non-physiological temperature and media, light exposure, mechanical handling, centrifugation or constant levels of CO_2_ and O_2_) that might modify AMPK activity. This figure summarizes the reported (beneficial or detrimental) actions in spermatozoa when extender media used in ART were supplemented with AMPK inhibitor (CC), AMPK activators (A769662 and AICAR) or indirect AMPK activators such as metformin and resveratrol (RSV).

As mentioned before, sperm capacitation is a mandatory process to fertilize oocytes and the events related with sperm capacitation (i.e.,: hypermotility, acrosome reaction, protein phosphorylation, etc.) consume large amounts of energy (ATP). It was also stated that AMPK acts as an energy sensor and it is activated under physiological and/or stressful sperm conditions. Therefore, there is a growing interest in this kinase to improve ART procedures where gametes are handled and subjected to different types of stress such as mechanical (centrifugation, pipetting), changes in temperature, levels of CO_2_ and O_2_, medium composition, extracellular matrix (solids, plastics), or to light exposure [109] (Figure 5).

### 6.2. AMPK as a Tool to Improve ART

The sector of animal farm is very interested in ART since livestock genetic background can be easily and successfully improved by the use of these techniques. Artificial Insemination (AI) was the first widely accepted ART for livestock. This technology allows genetically superior males to produce more offspring than it would be possible through conventional mating. More than 90% of western Europe’s pig industry performs AI using boar seminal doses preserved at 17 °C as a routine technique [110]. The temperature decrease during this type of preservation is aimed to reduce sperm metabolism, which subsequently leads to lower rates of both acidification and reactive oxygen species (ROS) production in the storage extender, both derived from accumulation of CO_2_ and lactic acid from oxidative phosphorylation and glycolysis respectively, obtaining the so called “quiescent spermatozoa”. Based on the principle that AMPK acts as a stress and energy regulator, it was reported that AMPK becomes activated when quiescent rat spermatozoa are subjected to decreasing temperatures down to 4 °C [61]. Several works have studied AMPK with the aim to improve ART when spermatozoa are stored under non-physiological temperature during semen preservation techniques. A study about boar seminal doses conservation at a non-physiological temperature (17 °C), demonstrated that before semen preservation (day 0), sperm AMPK phosphorylation was undetectable but sperm AMPK activity increased during the following days of preservation, reaching a maximum phosphorylation at day 7 [77]. Therefore, during semen preservation at 17 °C, at least in boar spermatozoa, the activity of AMPK fluctuates according to extracellular conditions that include stress, and is tightly regulated during the conservation period. This AMPK activation could be associated with a decrease of intracellular ATP levels described during boar sperm conservation [111,112]. Effects of different pharmacological drugs that influence AMPK activity (anti-diabetic compounds, such as metformin and rosiglitazone, or the inhibitor CC) during semen preservation, have been studied in different species [60,77,113]. In boar spermatozoa, only the percentage of motile spermatozoa was improved after a short preservation period (<4 days) when compound C was added to the extender [77]. However, other beneficial effects in semen quality were not found with the addition of CC [77] or metformin [113] to the boar semen preservation media. Both pharmacological approaches (metformin and CC) used during boar sperm conservation for 10 days cause similar negative effects: a reduction of sperm motility and lower mitochondria membrane potential [77,113]. Therefore, as mentioned before, any alteration of AMPK activity away from physiological levels during semen preservation leads to a detrimental effect on the main sperm functions [72,77]. These results in boar have been supported by a recent work in equine spermatozoa where compound C had a detrimental effect by decreasing sperm motility [60]. However, incubation of equine spermatozoa at physiological temperature with rosiglitazone, another antidiabetic compound that activates AMPK [114], increases the percentages of motile and rapid spermatozoa [60]. These authors suggest that rosiglitazone either protects and enhances mitochondria metabolism or induces a shift in stallion spermatozoa to a glycolytic metabolism (glucose uptake is increased), subsequently decreasing ROS and increasing intracellular ATP levels [60]. These positive effects in sperm motility led to authors to test rosiglitazone effect on stallion seminal doses preserved at 24 °C and results showed an improvement of equine sperm motility, suggesting its addition to stallion extender during preservation [60].

Sperm cryopreservation is other ART widely used that allows preserved indefinitely spermatozoa. Nevertheless, this procedure is harmful to the cells causing mainly structural damages to sperm membrane, DNA fragmentation and metabolic changes that restrict its fertilization chances [115]. These deleterious effects during cryopreservation are caused by cold shock, intracellular ice formation, oxidative stress, hypertonic damage, and combinations of these and others stressful conditions [115]. Many studies have tried to improve male gamete cryopreservation by adding antioxidants to the semen extender to reduce oxidative stress [116]. In this regard, the natural compound resveratrol that activates AMPK in some somatic cell types, when added to cryopreservation media, had positive effects on human spermatozoa [64,117]. Thus, AMPK was activated and ROS levels were reduced in human spermatozoa cryopreserved in the presence of resveratrol [64]. Moreover, DNA damage on human cryopreserved spermatozoa was ameliorated when resveratrol was added before cryopreservation, likely mediated by AMPK [117]. However, when resveratrol was added to boar seminal doses preserved at 17 °C, no improvement was detected in any quality sperm parameter analyzed, and negative effects were reported in mitochondria membrane potential, intracellular ATP content and motility [118]. Thus, the possible beneficial effects of resveratrol during semen preservation seem to be dependent on the species studied or the preservation protocol, where different actions of resveratrol might preponderate depending of the temperature, i.e., at −196 °C, the scavenging capacity preponderates, while at 17 °C, resveratrol negative actions might be due to Ca^2+^ mobilization over time. Besides mammalian spermatozoa, the effects of the classical AMPK activator AICAR or the indirect activator metformin were studied in avian spermatozoa. Thus, chicken spermatozoa cryopreserved in the presence of AICAR or metformin had stimulated sperm anti-oxidative defenses by partially restoring superoxide dismutase, glutathione peroxidase, and glutathione reductase activities, which subsequently decreased ROS levels and lipid peroxidation, leading to an improved sperm cryopreserved quality [119]. Also, in order to decrease oxidative stress, metformin was added (5 to 5000 µM) before mouse spermatozoa cryopreservation [74]. Although only the higher concentration of metformin activated AMPK, any concentration tested doubled sperm survival, motility, and percentage of motile spermatozoa, as well as the fertilization rate and embryo development without any reduction in membrane lipid peroxidation [74]. These results observed in mice are surprising since it is assumed that mitochondria is the major cellular target of metformin that leads to an inhibition of mitochondrial respiration [120]. In fact, boar spermatozoa preserved at 17 °C in the presence of metformin effectively had lower mitochondria membrane potential and also inhibited sperm motility [113]. Interestingly, the quality of cryopreserved stallion spermatozoa was not improved when AMPK activity was modified by any drugs tested: CC, AICAR, or metformin [59]. No significant improvement was observed in stallion sperm survival, total motility, progressive motility or live sperm with a structurally intact acrosome [59].

In general, the effects of the manipulation (up or down) of AMPK activity during ART protocols seem contradictory and are basically dependent on the pharmacological agent, species, and/or the ART protocol studied. As an example, whereas resveratrol did not have any positive effect during boar spermatozoa preservation at 17 °C [118], positive effects were described in cryopreserved human spermatozoa [64,117]. While metformin did not have any positive effect during stallion spermatozoa cryopreservation [59], very positive outcomes were described for cryopreserved mouse spermatozoa [74]. Moreover, it should be kept in mind that the effects of the different AMPK activators in spermatozoa from the same species vary according to the ART used. For instance, AICAR or metformin do not exert any beneficial effect in stallion spermatozoa cryopreserved for a long period of time at −196 °C [59], whereas the indirect activator rosiglitazone has some positive actions when spermatozoa are preserved a room temperature [60]. Therefore, we cautiously suggest that experiments designed with pharmacological agents that act through modification of sperm AMPK activity should be performed in each particular animal species and also under each specific conditions of ART used in order to address stress-associated problems originated by ART protocols and that ultimately lead to a decrease in sperm quality.

Effects of the AMPK inhibitor CC supplementation to sperm conservation media during ART protocols are described in Table 1.

As expected, negative effects were found in spermatozoa when the AMPK pathway is inhibited by CC such as reduced mitochondrial membrane potential (by 50%) and basal oxygen consumption (by 60%) in *AMPKα1* knock out mice [3], and detrimental effects on important sperm functions in refrigerated boar spermatozoa [77], human cryopreserved spermatozoa [64], chicken cryopreserved spermatozoa [119], or room temperature preserved stallion spermatozoa [60]. These negative effects can be summarized as: (i) decrease of mitochondrial membrane potential; (ii) sperm motility inhibition; (iii) loss of acrosomal membrane integrity; (iv) increase of plasma membrane disorganization; and (v) increase of apoptotic-like spermatozoa (Table 1).

Nevertheless, although differences were observed between species and depending on the ART used, in general, beneficial effects were found when AMPK was shown to be over-activated by metformin in cryopreserved mouse spermatozoa [74], rosiglitazone in stallion spermatozoa preserved at room temperature [60], AICAR in cryopreserved chicken spermatozoa [119] and resveratrol in cryopreserved human spermatozoa [64,117]. The positive effects observed were: (i) improvement of sperm survival, motility, and velocity; (ii) improvement of sperm antioxidant defense and decrease of lipid peroxidation (LPO); and (iii) improvement of embryo quality (Table 2).

However, this issue seems controversial, as negative effects have also been demonstrated, at least regarding metformin supplementation. Thus, sperm motility and mitochondrial membrane potential are negatively affected in boar spermatozoa preserved at 17 °C in the presence of metformin [113]. Above all, AMPK protein should be considered a suitable target to effectively improve assisted reproduction technologies’ outcomes where spermatozoa are subjected to different types of stress.

## 7. AMPK in Spermatozoa: A Physiological Link between Fertility and Energy Metabolism

The central role of AMPK in energy metabolism is well known, although its involvement in fertility control has only recently been pointed out. AMPK is present in female and male gonads and seems to contribute to the different stages of maturation of germ cells and spermatozoa by modulating hormone production (steroidogenesis) and their interaction with nourishing gonadal somatic cells. In mature mammalian spermatozoa, the ability to preserve energy homeostasis under fluctuating extracellular nutrients, physiological stimuli, or even stress conditions that occur through the female reproductive tract is a necessary characteristic of successful spermatozoa. Moreover, the energy charge-sensitive kinase, AMPK, performs an important and essential regulator of the main physiological processes of spermatozoa and therefore represents a proper target to improve assisted reproduction technologies. The increasing scientific evidence about AMPK in the male fertility area points out AMPK as the physiological link between reproduction and energy metabolism.

Moreover, considerable progress has been performed recently in the molecular development of direct AMPK activators/inhibitors. A future scientific challenge will be to integrate some of these promising therapeutic compounds derived from pre-clinical animal studies into practical improvements in the field of assisted reproduction in animals and human.

## Figures and Tables

**Table 1:** Effects of the use of AMPK inhibitor C (compound C) in spermatozoa functions of several species during Assisted Reproduction Techniques (ART).

Specie	ART/Stress	Sperm Effects	Reference
Boar	Preserved (17 °C)	1. At short period (<4 days): Improves the % of motile sperm 2. At long period (≥4 days): Decreases sperm MMP Decreases acrosome membrane integrity Increases plasma membrane disorganization	[77]
Stallion	Cryopreserved (−196 °C)	No effect described	[59]
Chicken	Cryopreserved (−196 °C)	Decreases motility and antioxidant capacity	[119]
Stallion	Preserved (RT)	Reduces sperm motility	[60]
Human	Cryopreserved (−196 °C)	Deleterious effect in sperm motility and mitochondria Increases apoptotic-like spermatozoa	[64]

**Table 2:** Demonstrated effects of direct (AICAR) or indirect AMPK activators (resveratrol, metformin and rosiglitazone) in the spermatozoa functions of different species during Assisted Reproduction Techniques (ART). Cryopreservation was always at −196 °C. * It has not been showed that the indicated compound effectively activates AMPK in spermatozoa from the indicated specie.

Compound	Specie	ART/Stress	Sperm Effects	Ref.
Resveratrol *	Boar	Preserved (17 °C)	Decreases sperm motility, MMP and ATP	[118]
Metformin *	Stallion	Cryopreserved	No effect described	[59]
AICAR *	Stallion	Cryopreserved	No effect described	[59]
Metformin	Mouse	Cryopreserved	Increases sperm motility and viability Increases fertility rate and quality of embryos	[74]
AICAR	Chicken	Cryopreserved	Protects against ROS and lipid peroxidation Improves motility and % rapid spermatozoa	[119]
Metformin	Chicken	Cryopreserved	Protects against ROS and lipid peroxidation Improves motility and % rapid spermatozoa	[119]
Rosiglitazone	Stallion	Preserved (RT)	Improves % motile and % rapid spermatozoa Shifts to glycolytic metabolism, increases glucose uptake and reduces ROS	[60]
Resveratrol	Human	Cryopreserved	Reduces ROS and apoptosis-like spermatozoa	[64]
Resveratrol	Human	Cryopreserved	Protects against apoptotic-like spermatozoa and MMP damage	[117]
Metformin *	Boar	Preserved (17 °C)	Reduces sperm motility and MMP	[113]

## Abbreviations

AMPK	AMP-activated protein kinase
ART	Assisted reproduction techniques
ICSI	Intracytoplasmatic sperm injection
IVF	In Vitro Fertilization
SC	Sertoli cells
LC	Leydig cells
sAC	Soluble adenylate cyclase
cAMP	Cyclic adenosine mono phosphate
PKA	cAMP-dependent protein kinase
PKC	Protein kinase C
LKB1	Liver kinase B1
CaMKKα/β	Ca^2+^-calmodulin dependent kinase kinases II
TSSK1/2	Testis specific serine/threonine kinases 1/2
AICAR	5-aminoimidazol-4-carboxamide-1-B-D-ribofuranoside
CC	Compound C or dorsomorphin
∆Ψm	Mitochondrial membrane potential
PMA	Phorbol 12-myristate 13-acetate
ROS	Reactive oxygen species
RSV	Resveratrol
